# “The hardest part of what we’re doing”: research staff perspectives on engaging marginalized populations in substance use trials

**DOI:** 10.1186/s13011-025-00657-7

**Published:** 2025-07-07

**Authors:** Kaitlyn Jaffe, Celia B. Fisher

**Affiliations:** 1 Department of Health Promotion and Policy, University of Massachusetts Amherst, 715 North Pleasant Street, Amherst, MA 01003 USA; 2 Department of Psychology, Fordham University, 441 East Fordham Road, Bronx, NY USA

**Keywords:** Substance use, Pragmatic trials, RCTs, People who use drugs, Qualitative, Research staff

## Abstract

**Background:**

Randomized controlled trials (RCTs) are a critical component of the development of pharmacological treatment options for substance use disorders. Pragmatic trials, in particular, aim to enhance generalizability by testing interventions in real-world settings. However, structural barriers, including socioeconomic marginalization and criminalization, continue to limit research participation among people who use drugs (PWUD). While prior research has explored perspectives of PWUD in research, less is known about how RCT staff navigate obstacles to engaging PWUD who experience structural disadvantage.

**Methods:**

We conducted semi-structured interviews with 36 U.S. research staff (i.e., research coordinators; research assistants) working on pragmatic RCTs testing the effectiveness of medications for substance use disorders.

**Results:**

Staff described challenges that complicated study enrollment and retention, including mistrust, negative perceptions of study components, restrictive eligibility criteria, and logistical challenges related to transportation, housing, and communication. Despite the more flexible design of pragmatic RCTs, staff still encountered constraints that conflicted with participant needs and necessitated going beyond their role to facilitate inclusion and retention. Research staff also identified important facilitators of recruitment and retention, including relationship building, leveraging referral systems, and adopting flexible, participant-centered approaches, where possible.

**Conclusion:**

Even in pragmatic trials designed for real-world conditions, social and structural disadvantages and rigid research structures can limit study participation among PWUD. Our findings suggest that with support, research staff play a central role in navigating these challenges and devising potential strategies for engaging marginalized populations in research.

**Supplementary Information:**

The online version contains supplementary material available at 10.1186/s13011-025-00657-7.

## Introduction

Randomized controlled trials (RCTs) are a rigorous method of hypothesis testing interventions but are challenging to implement for trial staff. Challenges include balancing clinical and research priorities, recruiting sufficient participants, negotiating equipoise, and working collaboratively [1–5]. Investigation around these challenges has been conducted across clinical fields, including oncology, mental health, and HIV, but few studies have examined experiences of staff involved in substance use research. Substance use trials play a critical role in addressing the ongoing overdose crisis and unmet treatment needs of people who use drugs (PWUD) [6, 7]. For instance, the NIH HEAL (“Helping End Addiction Longterm”) Initiative has invested $2 billion in opioid-related research and enhancing RCT infrastructure to investigate substance use treatments [8]. As these trials expand in scope and scale, it is critical to examine the practical and ethical complexities of conducting research with populations that experience structural vulnerability and social marginalization.

Substance use research is particularly complex, given the interplay of issues specific to some drug use. PWUD may experience health comorbidities and wide-ranging social and structural disadvantages (e.g., unstable housing, violence, criminalization) that they report as barriers to participation [9–14]. Further, experiences of substance use disorders are variable. Drug use frequency, mode of administration, drug profile/composition, and polysubstance use can vary within and between participants [15]. Such heterogeneity can make standardization of research protocols difficult, creating implementation challenges or prompting investigators to exclude large swaths of participants altogether [16–18]. Recruitment and retention are further complicated by the legal, social, and moral milieu surrounding drug use. Problematic drug use remains stigmatized among the public and healthcare providers, with stigma extending to substance use treatments [19]. Previous work has explored how opioid agonist treatments are framed as “dependence” or illegitimate among providers, the public, and PWUD—perceptions that can deter PWUD from seeking treatment or enrolling in research [20–24].

Given these potential issues in substance use research, it is important to recognize how traditional RCTs can exacerbate such challenges. While RCTs are considered the “gold standard” for clinical research, they have been critiqued for their lack of external generalizability and exclusionary practices, particularly of marginalized groups [25]. Previous research has documented that people of lower socioeconomic status, people of color, people with mental health issues, and people experiencing homelessness are frequently underrepresented in clinical research [25–27]. These populations may be excluded due to trial characteristics (e.g., strict eligibility criteria), stuctural barriers (e.g., lack of transportation), or their study perceptions (e.g., justified medical mistrust rooted in histories of research exploitation) [11, 16, 28–32]. Ultimately, these patterns of exclusions may result in epistemic bias, where the knowledge generated is not representative or generalizable to all populations [33].

Pragmatic RCTs, a type of comparative effectiveness research [34], are increasingly used to address these issues of generalizability by evaluating the effectiveness of interventions in the “real-world” [35–37]. These trials often compare two approved treatments, treatment versus no treatment, or alternative approaches to care delivery (e.g., dosing, administration, regimens). Unlike traditional RCTs, pragmatic trials aim to reflect actual conditions, using more flexible protocols and enrolling more diverse participants to accurately reflect the population who might receive the intervention [34, 38]. However, challenges around pragmatic trials persist. Conducting trials in real-world healthcare settings does not resolve the underrepresentation of marginalized groups who experience barriers to accessing healthcare [1, 38]. In addition, scholars have also problematized the blurred boundaries between research and healthcare when trials are embedded in healthcare systems, as well as the logistical barriers to nesting research in clinical care processes [1, 39–41]. Despite their promise, pragmatic trials may still reproduce some of the same challenges seen in traditional RCTs and with margainalized people who use drugs.

While much has been written about the challenges of recruitment and retention in clinical trials, most research focuses on participant-level challenges, hypothetical willingness to participate, or study design features that influence enrollment (e.g., stipends, study burden, or recruitment marketing) [42–45]. Fewer studies have focused on perspectives of research staff, who face these challenges directly, particularly within pragmatic trials embedded in healthcare settings [39]. By centering the experiences of staff operating within RCT structures, this study offers a critical window into how structural and institutional dynamics shape recruitment and retention and how staff navigate these challenges to conduct effective research with PWUD.

## Methods

### Study design and eligibility

To explore the complexities of conducting substance use RCTs, we sought to understand the perspectives of research staff, an objective best suited for qualitative methods. We chose semi-structured interviews over focus groups to understand social processes as well as individual variation in staff roles, responsibilities, and experiences, while also ensuring confidentiality to avoid participant perceived risks to employment [46]. To enhance the study’s relevance and rigor, we engaged a community advisory board (CAB) comprised of 10 individuals with either direct experience working as research staff in substance use RCTs or with substantive or methodological expertise in the ethical dimensions of RCTs. Meetings took place for 1.5 to 2 h over Zoom, where CAB members provided extensive feedback on the eligibility screener, recruitment strategy, and interview guide.

Participants were eligible for this study if they had worked: (1) on an RCT in the past 5 years; (2) with people with opioid or stimulant use disorders; and (3) in trials focused on testing medications for feasibility, acceptability, or effectiveness (i.e., pragmatic trials testing established medications with new formulations, updated protocols, in novel settings, etc.). We focused on pharmacological and not behavioral protocols in order to capture the complexities of medication regimens, participant expectations, and regulatory constraints that may not be as salient in non-pharmacological studies and for greater consistency across research staff experiences. We were interested in perspectives of staff engaged directly with PWUD, and so we limited eligibility to research assistants and coordinators. Research assistants and coordinators are tasked with recruiting, enrolling, and following up with participants and they possess first-hand experiential knowledge of the day-to-day practices and challenges that arise within trial settings. At the same time, they serve as “key informants,” offering interpretive insights into broader structural dynamics at play, such as the marginalization of PWUD, but where they may not be direct observers [47].

Participants were recruited through a national email listserv of research coordinators and assistants in substance use RCTs, apart from four participants recruited via professional networks. Potential interviewees were directed to a Qualtrics link, where they received study information and completed a brief eligibility questionnaire. These questions asked about research experience, study role, basic demographic information, and interview availability. The study was deemed exempt (Category 2) from review by the university’s Institutional Review Board (HUM #00225512).

### Data collection

Interviews were conducted one-on-one over Zoom between April and May 2023. Prior to the interview, participants received study information, which was reviewed at the start of the interview. Interviews lasted an hour, and participants received a $60 gift card. Interviews focused on respondents’ professional backgrounds, current role, study experiences, training, collegial relationships, and perceived support. Interviews were audio-recorded, transcribed, and cleaned. Any potentially identifying information was removed from the transcript.

### Data analysis

Our analytic process was informed by a constructivist perspective, with the assumption that knowledge is co-produced in interactions between the interviewer and participant [48], just as they are between research staff and participants in RCTs. We sought to attend to both the practical realities of day-to-day study implementation as well as the social meanings that research staff ascribe to their experiences. In this vein, we pursued an abductive approach, moving back and forth between participant-generated insights and existing knowledge related to recruitment and retention, versus a purely inductive or deductive approach [49]. Transcripts were analyzed using a multi-step “flexible coding” strategy [50], a pragmatic, iterative method of analysis. First, using Dedoose software, data were indexed by the first author into broad categories that mirrored the structure of the interview guide. Categories were later clarified with discussion between authors. Next, indices related to recruitment and retention were broken into smaller analytic codes (themes) and applied to the transcripts. Finally, Dedoose excerpts and chart tools were utilized for validation and to understand thematic saturation [50]. See Supplementary Table 1 for examples of interview questions, associated codes, and exemplar quotes.

## Results

In total, 36 research staff were interviewed. Staff worked in research sites across the U.S., with 19 (52.8%) in an academic medical setting and 17 (47.2%) in community-based treatment clinics, with research oversight from local universities. In the eligibility screener, respondents identified their gender, race, and ethnicity (all open-ended), and level of education. Participant demographics are displayed in Table 1. In general, respondents described current or previous work on NIH-sponsored studies comparing pharmacological treatments for substance use disorders, to assess effectiveness in real-life settings (i.e., pragmatic trials). While study objectives varied, respondents described engagement in similar trial processes (e.g., randomization, follow-up visits, treatment administration), strategies for recruitment and retention (e.g., payment for participation), and patient populations. Many research staff, especially in academic centers, were assigned to multiple studies, and experienced staff drew upon both current and previous study experiences in their responses.

**Table 1:** Characteristics of the study sample, 2023 (*n* = 36)

Characteristic	*n* (%)
**Gender**	
Women	26 (72.2)
Men	8 (22.2)
Non-binary	2 (5.6)
**Race and Ethnicity**	
Asian	1 (2.8)
Black, African American	3 (8.3)
Hispanic, Latino/a^**†**^	7 (19.4)
Mixed race	1 (2.8)
Native American	1 (2.8)
White	23 (63.9)
**Education**	
Some college; Associate’s degree	3 (8.3)
Bachelor’s degree	19 (52.8)
Master’s degree	12 (33.3)
MD/PhD	2 (5.6)
**Study Role**	
Research Assistant	12 (33.3)
Research Coordinator, or similar title	24 (66.7)
**Study Site**	
Academic setting	19 (52.7)
Community setting	17 (47.2)

^**†**^ Open-text responses included “White and Hispanic,” “Hispanic”, “Latino/a” and “Puerto Rican”

### “A lot of distrust and uncertainty”: barriers to recruitment

#### Social and embodied experiences of drug use

In recounting their trial experiences, staff described recruitment as the most difficult aspect of research, “literally the hardest part of what we’re doing, honestly” (Staff 12). Beyond the typical RCT pressures, some aspects unique to drug use made recruitment interactions especially hard. For instance, some staff found difficulty in effectively describing the study to potential participants who were starting to experience withdrawal during the visit: “if someone’s in withdrawal, they’re feeling terrible, and you’re going through a 30-minute [screening] survey with them. It’s bad for everyone” (Staff 9). Some trial protocols required that participants stop using substances before initiating study medications, given the risks of precipitated withdrawal (i.e., medication-induced, rapid withdrawal), which could deter participants. While most PWUD were interested, staff had to contend with embodied effects of problematic use, navigating participants’ physical discomfort and hesitancy throughout enrollment.

Substance use stigma also shaped recruitment through study advertisements, study referrals, and among prospective participants. Several research staff described challenges in advertising for a drug-related study (e.g., social media, flyers, bus ads):

There’s keywords you can’t use [in advertisements]. If we’re recruiting for a meth study, I need to say the word “meth.” And so if I can’t say the word “meth,” regardless of what’s around it, like, “do you wanna quit meth?” We can’t use a lot of advertising outlets and we’re in the South, so things are pretty strict still in regards to certain words and things like that. -Staff 16.

Such vague advertising could hinder recruitment efforts and attract people who were ultimately ineligible. Trials also relied on referrals from healthcare providers, who might disagree with study aims or feel hesitant in broaching the topic with patients. One research staff observed, “I think there’s a big dose of stigma [among referring providers]. The idea is like, ‘I don’t wanna make my patients mad. I just kinda wanna go through the day and I don’t wanna ruffle any feathers’” (Staff 30). Staff also raised the issue of internalized stigma among prospective participants, related to substance use, study medications, or fear of disclosure. While some trials were embedded in large medical centers, where study attendance would be less conspicuous, other sites were in smaller town clinics, with greater risk of disclosure:

There was a large challenge in terms of even getting new patients in, just for treatment in general, not even with the study alone, because of the stigma in these really small communities. We’d often hear that people would park multiple blocks away from the clinic, because everyone in town knew what time a group session was happening. And so, if they saw your car, even at the dollar store lot next door, they might assume, “Oh, I’m in treatment.” And so there was a lot of stigma around that. I think that was a bit challenging in terms of getting our numbers for the study. -Staff 26.

As staff highlighted, substance use stigma played a significant role in study enrollment, reflecting a recruitment landscape unique to stigmatized conditions.

#### Study perceptions

Beyond stigma related to drug use itself, staff also discussed how broader perceptions and misunderstandings about the study shaped willingness to enroll. Respondents described mistrust among PWUD related to negative healthcare experiences or around confidentiality, given the criminalization of substance use. Responding to mistrust comprised a substantial part of recruitment efforts: “As we recruit, there’s been one thing we’re kind of working against is there’s been a lot of distrust and uncertainty. We get questions like, ‘Are you going to share my information with authorities, if I tell you I’m using a substance? Are you going to check my ID if I don’t have a proper ID?’” (Staff 12). Mistrust also extended to study participation: “Sometimes participants hear ‘research,’ and they think ‘guinea pig,’ like automatically. So, we have to reiterate, ‘no, that’s not what’s going on here’” (Staff 6). Conversely, some perceptions reflected participants’ lofty expectations, anticipating a panacea:

A lot of them come in here thinking that we’re testing some kind of miracle cure, that we’re testing something brand new, it’s groundbreaking, you know, [that] we guarantee that you’re gonna get better. No. We don’t guarantee you get better. And you can see there’s a little bit of pause, because I think a lot of them are looking for something…They see the terms “research” and “opioid use disorder”, they’re like, “oh, hey, they’re onto something new that we could potentially benefit from.” Well, you can, but we’re not guaranteeing that. -Staff 8.

Since these pragmatic trials were testing established treatments, staff noted some prospective participants had familiarity with the medications, which could influence enrollment decision-making. For instance, staff remarked that some participants had concerns about the dosage being either too high or too low based on previous experience. Others were apprehensive about medication administration, especially participants with needle anxiety, substance use triggers around needles, or discomfort about forgoing the daily ritual of oral medication:

We’ve had others that started—were randomized to the investigational [injectable] product and they maybe received a couple of injections, but they found that their recovery was very dependent on the ritual of taking their medication every single day at the same time. That’s really common, we hear that a lot, and so switching up their routine oftentimes was like they found it to be detrimental to sort of…their psyche behind their recovery, and so a lot of them have switched back to their standard of care [oral medication]. -Staff 36.

As their studies progressed, staff could anticipate these concerns and devised effective strategies for addressing them during enrollment. This included reassuring confidentiality, clarifying details, and sharing anonymous participant testimonials with prospective participants.

#### Study eligibility

While staff addressed participation barriers, they felt that some participants were excluded due to overly strict eligibility criteria, including age, substance use patterns, mental health comorbidities, hospital admissions, pregnancy, housing, and being in residential treatment. Some expressed concern that criteria would exclude those experiencing socioeconomic marginalization. For instance, some studies excluded people without a phone: “With the study that I run into, it’s people are missed most of the time because they don’t have a phone, and they would otherwise be an excellent participant, but we just have no way of contacting them or achieving that eligibility” (Staff 15). Other staff felt people were inadvertently excluded, including those without transportation or those outside the clinic patient roster from which the study recruited. Across trials, staff grappled with the potential exclusion of a population defined as “vulnerable” by research institutions, such as one staff who spoke about the inherent exclusion of participants with criminal-legal system involvement (e.g., drug court), reflecting a broader “stigma-vulnerability nexus” in policy, wherein PWUD are framed as simultaneously “at-risk” and “risky” [51]. This study screening process could be disheartening for both staff and prospective participants, as they underwent lengthy procedures only to be deemed ineligible, with few other treatment options (e.g., treatment deserts; no approved treatments for stimulant use disorders). As one respondent described, “Especially with substance use trials, there’s so much screening you have to do, it can be brutal and emotional, and if you’ve built this connection with someone and then you have to tell them, ‘no, you can’t proceed to the majority of this study.’ It’s hard. It’s very sad” (Staff 29). Some staff acknowledged that during recruitment, these emotional and social demands took priority and they often linked prospective participants to services, even if they did not enroll: “Whether that’s they need to get connected to mental healthcare or housing or something…Sometimes it’s just not the right time and I’d rather help you get settled and stable and then when you’re ready, we can worry about research” (Staff 1). As this respondent illustrates, many staff were not only recruiting participants and assessing eligibility, but contending with their social, emotional, and physical needs.

### “There’s no judgement here”: facilitators of recruitment

#### Establishing trust

In recognizing the criminalization and healthcare discrimination faced by PWUD, staff emphasized how they fostered a welcoming environment, which helped lay the groundwork for trust with participants. Staff were attentive to their self-presentation and emphasized a distinction from clinicians: “People in this population tend to have a lot of mistrust towards healthcare or have had negative experiences in the past with healthcare. The more we identify ourselves as research or the further away from clinical care we make ourselves, I think in some odd way that makes people really comfortable sharing things with us” (Staff 15). Some staff disclosed their lived experience with substance use to build trust:

I haven’t forgotten where I came from. I may be 15 years clean, but I still remember sitting in that chair and in that jail cell, and I was just so sad and lost and scared. And so for me, remembering that, helps me to let them know like, “Hey. I understand, I get it, I totally get it. There’s no judgement here,” because the moment they feel like you’re judging them…you lose them right there, and that trust is not gonna come back. -Staff 4.

Staff also emphasized transparency and the voluntariness of the trial and that they were collecting data without judgement, further distancing themselves from treatment facilities where positive drug screens have consequences. As one staff emphasized, “These people need to know when they walk in—and you can see them, and they’re hanging their heads down, they’re like, ‘look, I used this past weekend.’ That’s okay. That’s data. This is observable. This is not a pass-fail scenario. No one’s gonna come in here and judge you and kick you out of the study” (Staff 8). Despite the importance of these trust-building and emotional support practices, this work may not be formally recognized in research protocols and instead improvised through the course of the trial.

#### Referral sources

When asked what made recruitment easier, many staff cited their referral systems, particularly those embedded within treatment clinics. These research offices were located near the clinic or utilized automated systems where clinicians could refer patients. While staff identified barriers in establishing these connections and generating initial support from clinic staff, overall, these relationships and systems boosted recruitment. As one staff explained,

We can recruit directly from our detox program. So if someone is calling in for services, the call center has their script of—it’s a form that they’re working through, and there are two questions that will get them routed to us: “are you interested in a research program?” and “no cost—” and I think they list the two medications that we offer. We get probably 80% of our referrals that way, and that pretty much keeps us afloat. -Staff 10.

Research staff embedded in healthcare settings could also rely on clinic resources, including social workers, clinicians, and programming, when prospective participants were ineligible for the study and needed referrals.

For sites without in-house recruitment, staff had to build connections with broader communities, including advertising at community centers, social service venues, and events. Some staff remarked that since the pandemic had shuttered many recruitment sites, they tried more creative, community-specific outreach strategies:

We were out in the community for hours, going into food banks and talking to them and asking if we can put up flyers, going to local bars and talking to bar owners. Some people were with it, some people were like, “Absolutely not.” Local libraries. I remember we went to a dental clinic that would have populations that would come in who, you know, people who had methamphetamine use and had dental issues. They were so happy to have us there. -Staff 13.

Some sites employed “recruitment and retention specialists,” or staff with community connections who spent their time in the field recruiting for studies, a strategy that interviewees deemed effective. A few staff also reported working with professional marketers or recruiters to design and place advertisements, which could be effective but costly.

### “A needle in a haystack”: barriers to retention

Once participants were enrolled, staff faced the complex task of keeping participants returning for study visits and adherent to treatment. Staff described the central challenges in longitudinal research with this population and key strategies that made retention easier.

#### Socioeconomic marginalization and inflexibility

As highlighted, PWUD often experience socioeconomic marginalization, which could hinder regular study visit attendance. Transportation was an issue, especially in rural areas without public transportation or rideshare services that staff could arrange. Even if not an explicit eligibility criterion, transportation access could shape recruitment and retention and ultimately, the study sample:

The county that I live in is still pretty segregated by design from decades and generations ago. And that is actually reflected in our population…most of our population uses public transportation or just walks here. And so if you are not able to come to the building, then you’re not going to come from [A] County to [B] County. -Staff 10.

Even in locations with public transit, staff described buses as potentially unreliable, prompting issues with attendance or medication pick-up. For instance, one staff recalled a participant’s situation: “The bus was late, so then they were late, and then that caused issues with getting their take-homes [medication] and they had to basically start over to earn the trust to get their take-homes. And it’s like, ‘but it wasn’t my fault, the bus was late’” (Staff 26). In this example, the participant had attended regularly enough to receive a more flexible but regulated form of the study medication (“take-homes”) but being late or missing a dose was enough to rescind their access. While staff attributed some of these challenges to socioeconomic marginalization, these moments also underscored how rigid study protocols may inadvertently penalize participants for circumstances beyond their control and reinforce hierarchical dynamics. Housing instability also prompted issues for safe storage of study medication and for contacting participants:

[This city] has such a huge homeless population, I would say probably maybe 65% of our participants are unhoused and it’s just hard to locate people. We do things like say, “if you call us and update us on your new phone number, we’ll give you $5.”…We have tried to go out. We have a van that we stock with socks and snacks and water and all the things, and we’ve tried to go out and just kind of find people and that was a terrible failure, like a needle in a haystack. -Staff 20.

As staff struggled to retain socioeconomically marginalized participants, they might ultimately shift their recruitment strategy to focus on easier-to-reach participants. These subtle shifts in recruitment likely improved retention rates and reduced study disruptions but could shape participant pools dramatically. Several staff described selection effects:

[The participants] have it together a lot more than the population [of PWUD] usually does in general. I don’t mean that in a bad way at all, but they just seem to have more resources, live in more convenient locations, have more transportation, financially better off than in general. And that makes it easier. I think that’s also why they volunteer to participate in the study. They have a little more time, their lives aren’t so chaotic, they can handle it. -Staff 11.

Another staff reiterated, “Our studies are a little bit self-selective in that they’re already women who were going to be successful in recovery anyway, and that’s what’s made them successful in the study” (Staff 36). These insights suggest that even in pragmatic trials, designed to enhance generalizability, the pressure to retain participants may incentivize the exclusion of less “reachable” or “stable” participants, further reinforcing structural inequities around representation in research.

#### Scheduling and staffing

Some staff also cited scheduling challenges as a barrier to retention. One staff explained: “A lot of the day-to-day is coordinating visits because this patient population is a bit difficult to keep track of in certain regards. There are a lot of no-shows, a lot of last-minute rescheduling. Sometimes people show up early by several hours, sometimes people show up late by several hours. So there’s a lot of that kind of coordination that eats up some time” (Staff 33). Further, most sites were only open from 9am to 5pm, creating difficulty for participants with work or other commitments, and making staff unavailable outside the study hours. One respondent described their dilemma in managing a balance:

I would become really protective of all of these participants’ safety, and I took that very seriously. And so, we had study phones that [participants] would call if there was an issue, but oftentimes they would call when we had already gone home. So then it’s, “well, maybe I should take the study phone home, so if there’s an emergency, I can be contacted.” But then you have other people in the office being like, “but you’re not being paid to do that. You shouldn’t have to answer the phone at 9:00 o’clock at night, if there’s something going on, that’s not your job.” But no one else is stepping in to do that, like the provider, the PI at the clinic hasn’t thought to step in to do that and they’re not willing to do that. -Staff 26.

As this quote illustrates, there may be ethical tensions when research staff’s sense of responsibility exceeds the expectations of their job. Beyond the restricted hours, some staff found difficulty in scheduling participants with limited staff capacity or amidst high research assistant turnover. Certain study visits were limited by the availability of clinicians, as the only staff who could conduct medical assessments: “We’ve also had issues hiring any kind of clinician with whatever title or degree for whatever reason. That’s really impacted our ability to schedule appointments. [The appointments are] having to happen at really specific times, it’s not 40 hours a week, it’s like 15 or 20 probably” (Staff 12). While study staff might have anticipated participant-related retention barriers, site and staffing issues interfering with retention efforts were unforeseen challenges.

### “They knew our faces”: facilitators of retention

#### Building a positive research environment

While establishing trust during recruitment encouraged enrollment, staff needed to maintain positive relationships to support attendance. Nearly all staff recognized that study stipends encouraged follow-up attendance, but felt their relationships with participants also facilitated retention, as participants seemed to experience a secondary benefit of these prosocial interactions. One staff remarked, “I think our participants get very used to seeing us each week, coming in and interacting with the staff, and sometimes people are like, ‘This is the only thing that I do outside of work.’ So sometimes with the follow-ups, they look forward to just coming into the office” (Staff 28). A few staff noted the importance of researcher continuity: “I do think they enjoyed that piece, just knowing that somebody was looking forward to them coming…They knew us, they knew our faces, they knew our names. It wasn’t someone new always that they had to get to know or explain their story to all over again” (Staff 13). As during recruitment, almost all staff described how they approached study interactions with respect and a desire to learn about participants (e.g., remembering birthdays and personal details). A handful of staff wondered whether building such connections was disingenuous or fell outside the scope of research, more like a therapeutic relationship. One staff explained:

The research team is doing things that might not typically be seen as appropriate for research. Like there’s definitely a different set of boundaries with the resources provided and sort of the befriending that needs to happen. It just doesn’t really take into account that honestly being these women’s friends is part of what is necessary to make this study successful. It’s been something I’ve kind of had to make up as I go. And sometimes I feel like, “Am I gonna get in trouble for this?” Like, no, because my retention numbers are so good, but also it’s kind of like the lines are really blurry with these populations. -Staff 36.

Staff also spoke about the intentional warm and welcoming spaces they created within their sites that likely boosted retention. One researcher described the slower pace of research, compared to the clinical side of the facility: “We don’t have to move people in and out quickly. That is not how the study is set up. So if they’re going to be with us from anywhere from like 40 minutes to four hours, then let it be an enjoyable experience” (Staff 10). Some research staff framed the site as a refuge: “Some of [the participants] are displaced people and so just coming to the clinic is giving them time away from being outside…They have told us, ‘I enjoy coming here, but at the same time I’m also here ‘cause I’m not outside for a few hours’” (Staff 32). Staff described the amenities they provided, including coffee, childcare, food pantry, clothing closet, tents, tarps, and sleeping bags—some of which are critical items for unhoused participants. While staff emphasized how the sites differed from clinical environments and fostered retention and engagement, these examples show how relational and material supports are essential to the success of a study, beyond what might be dictated by the trial protocol.

#### Flexibility

Finally, staff emphasized flexibility in study retention. At times, they had to be resourceful or go beyond their study roles: “Anytime we have a participant that winds up in jail—and that has happened—we still get the flexibility of going to the jail and still collecting data, which I think helps. It becomes less exclusionary that way because if you said, ‘well, they can’t make it to the study site, they’re out,’ you’re gonna limit that window very much” (Staff 8). While most studies exclude people who are incarcerated due to their federal status as a “vulnerable population,” this study may have worked with their ethics board to gain approval to retain this participant—a retention strategy reflecting inclusivity and flexibility. If participants had transportation barriers, staff could pick them up or arrange rideshares. Other staff would meet participants at home:

There were some times when people would miss appointments, like, “My car broke down. I can’t do it. But I really wish I could be involved.” So we would like pack ourselves in the car, [with the] coordinator, someone who’s driving, somebody who can administer the medication, we would just take the study to them. So that they can stay in the study and we wouldn’t break protocol. -Staff 13.

However, some respondents highlighted how this flexibility was only possible for staff with greater availability, including those without children:

I’m also really lucky because that medical clinician, me and my other main coordinator, we’re all childless, which better or worse, it makes it so that we can stay late, we can…We are extremely flexible. Like I have another coordinator—she’s great and she gets the job done and she does everything, but she has to leave at a certain time to pick up her kiddo from daycare, and this population does not abide by regular times, which can be really frustrating. -Staff 36.

Collectively, these narratives underscore how effective strategies for retention, including relationship building, flexibility, and logistical support, stem from the experience and willingness of research staff and highlight potential discrepancies between trial protocols and infrastructure and the needs of marginalized PWUD.

## Discussion

This study explored research staff’s perspectives on recruitment and retention in pragmatic substance use trials. While recruitment and retention challenges are well-documented across clinical research fields [52, 53], our interview data highlight how these issues are shaped by the specific social and structural context surrounding substance use (see Supplemental Table 2 for overview of recruitment and retention challenges). Staff insights highlight how even pragmatic trial protocols can limit participation among the most marginalized and how staff adapted to these constraints. We situate our findings within existing literature on engaging marginalized populations in research, the limitations of RCTs (including pragmatic trials), and their epistemic tensions, with implications for future pragmatic trials with PWUD.

Previous research has documented how stigma and socioeconomic marginalization shape research participation, including willingness to participate, trust in research, and retention barriers [11, 25, 29, 45]. Our findings extend this work by demonstrating how research staff mitigated these barriers. Staff supported participants’ material needs (e.g., transportation, childcare) and connected both enrolled and ineligible participants to other services. Beyond this, staff also supported participants’ emotional needs. Staff built rapport and engaged in what one staff called “befriending” in clinical settings where participants might face institutional mistrust and isolation. While this relational labor seemed to enhance retention, as reflected in respondents’ narratives, it could also complicate the role of research staff. Such practices echo literature on the dilemmas of offering individualized care in other trial populations [3] and speak to broader questions of ancillary care obligations within inequitable contexts characterized by socioeconomic deprivation, limited access to effective treatments, medical mistrust, and access to a potentially fatal drug supply [54, 55]. Some staff embraced this role and forms of care while others experienced discomfort, reflecting tensions between the demands of study protocols and the realities of research practice.

While pragmatic trials address many of the limits of traditional RCTs by incorporating greater flexibility and reflecting real-world settings to increase generalizability, protocols may still be at odds with participant needs. For instance, staff recalled that participants transitioned from daily oral medication to long-acting injectables perceived this as a loss of a stabilizing ritual and highlighted how protocol design may ultimately undermine participant autonomy. As prior work has shown, trial participation can reorient PWUD’s spatial, social, and behavioral patterns in ways that complicate their treatment engagement [12]. Our findings further revealed that pragmatic trials can still replicate many of the same structural exclusions as traditional RCTs [17, 18]. Socioeconomic barriers such as lack of housing, transportation, and phone access regularly impeded recruitment and retention, and yet study structures remained inflexible. For instance, missing a dose due to public transit issues could ultimately penalize a participant, undermining their autonomy and trust and discouraging future research engagement. Moreover, staff described a subtle tendency to favor participants who were easier to recruit and retain, inadvertently creating a sample comprised of people with less intensive substance use and greater overall stability. While not necessarily protocolized, this practice reinforces exclusion of “vulnerable” groups, a pattern reflected across clinical research [56], thereby shaping study populations in ways that undermine generalizability of trials aimed at addressing substance use [16–18, 57].

Our findings suggest implications for future trials. First, participant-centered supports, including transportation, phone access, and flexible scheduling, should be built into trial design and budgeting from the start. Researchers may have both a professional and moral obligation to provide ancillary care, forms of care for participants that are “not required to make a study scientifically valid, to ensure a trial’s safety, or to redress research injuries” [58]. While many staff went above and beyond to provide these supports, a lack of institutional backing may create variability in implementation and risk staff burnout or role conflict. Second, investigators should explicitly consider how recruitment and retention strategies can reduce structural barriers to participation without placing undue burden on frontline staff. One strategy may be to utilize “recruitment and retention specialists,” as staff highlighted, who have built strong relationships with marginalized communities and may be more attuned to their needs. Finally, ethics training and supervision structures should acknowledge and support the emotional labor that staff perform in trials involving marginalized groups.

### Limitations

This study has limitations. First, interviewees were largely recruited from an NIH email listserv, a funding body that supports more complex, multisite trials. Staff operating smaller trials may face different challenges. Nonetheless, many U.S. substance use studies are NIH-funded, so it is likely that these respondents’ experiences reflect many staff operating RCTs. Second, research staff offered valuable insights from first-hand study experience operating a trial, but their perspectives as “key informants” are interpretations of the experiences of PWUD in research, rather than direct accounts. Third, given the small sample size typical of qualitative studies, we did not analyze results by staff demographics. Demographic characteristics and lived experience may shape perspectives, especially in participant interactions. Finally, we did not analyze results by trial characteristics, treatments, or topic of inquiry.

## Conclusion

This analysis illustrates the tensions that research staff navigate when recruiting and retaining marginalized populations in pragmatic substance use trials. Although addressing the broader context of marginalization falls outside the scope of pharmacological research, staff worked to bridge gaps by building relationships, demonstrating flexibility, and offering care ancillary to study aims. The insights gleaned from frontline staff reveal both the structural limitations and potential of RCT research with PWUD, and underscore the need for supportive trial infrastructure to produce generalizable results that expand treatment options across populations and contexts. Future research could build on these insights to examine how staff manage ethical boundaries and role expectations, how trial protocols shape patterns of inclusion and exclusion, and how integrating participatory approaches into RCTs could reorient study design toward greater equity in substance use research.

## Electronic Supplementary Material

Below is the link to the electronic supplementary material.

Supplementary Material 1

## Data Availability

No datasets were generated or analysed during the current study.

## Abbreviations

NIH: National Institutes of Health
RCT: Randomized controlled trial
PWUD: People who use drugs
