# Supplementary material for: “The hardest part of what we’re doing”: research staff perspectives on engaging marginalized populations in substance use trials
Source: Subst Abuse Treat Prev Policy. 2025 Jul 7;20:28. doi: 10.1186/s13011-025-00657-7 (PMC12236004; doi:10.1186/s13011-025-00657-7)
Supplement: Supplementary file 1 — Supplementary Material 1 [file 13011_2025_657_MOESM1_ESM.docx]

**Supplementary Table 1: Examples of interview guide questions**

| **Interview Question** | **Exemplar Quote** | **Associated Code** |
| --- | --- | --- |
| What has made recruitment easier for you? | “We've been around for about a year now, actively recruiting, the detox staff are very familiar with us. So when they get someone new, they're happy to call us and let us know this so we can go over and talk to them, that's been really nice. We've started reaching out to other departments, like our outpatient counseling. They're now in contact with us 'cause they're learning more about us, so that's also helped with recruitment.” | *Recruitment facilitators > Linkage with health and treatment services* |
| What were some challenges you encountered? | “The other study that I do works with postpartum women with methamphetamine use disorder, and that one is hard because one of the criteria they have to be self-reported no use for four weeks, which for a lot of women is... A lot of them four weeks is a huge milestone for them in recovery to meet and very often we couldn't get them. They couldn't get the full four weeks before being out of window, so... Yeah, I'd just say compliance and sort of sustained sobriety have been two of our hardest [challenges].” | *Recruitment barriers> Study eligibility criteria*  *Recruitment barriers > Substance-use related barrier* |
| What part of follow-up visits did participants enjoy or look forward to? | “Paid visits… It's not just that though… sometimes we're the only person that the [participants] come into contact within a week or every two weeks or every month that, like, listens to them and is nice to them and we try to make a real comfortable area and we've got all kinds of good snacks and sodas and coffee and [we] just treat people like human beings. So, I think some people enjoy coming in and being able to talk to someone on just a human level and be treated with respect and care. And you can see that with them 'cause they'll talk for a really long time and that's great because they're comfortable.” | *Retention facilitators > Study environment*  *Retention facilitators > Positive interactions* |
| What part of follow-up visits did participants not enjoy as much? | “We really don't have cabs and Ubers here. We are considered a metropolitan area, but we are tiny compared to other cities. The bus isn't great either, but we do provide them with bus passes that are free though. We try to—I have picked participants up. My other staff has picked participants up. We have some applications and documentation where they can get reimbursed through their insurance for their travel. We try our best to compensate for every study that we do just to help them. If nothing else is gas money, even though fewer and fewer people are using it for gas money 'cause gas is just so expensive.” | *Retention barriers > Socioeconomic marginalization*  *Retention facilitators > Flexibility* |
| What were some strategies you used to develop rapport with participants? | “We made sure to even think about like the way we dressed, which was very different than the other people at the academic medical center because of our population. We wanted to be more personable so we took into account all of these things to make sure that they had the best experience that they could.” | *Retention facilitators > Positive interactions* |

| **Supplementary Table 2: Barriers and Facilitators to Recruitment and Retention** |
| --- |
| **Recruitment** |
| ***Barriers*** |
| Social and embodied experiences of drug use  *Experiencing withdrawal during study screening*  *Substance use stigma in advertising, referrals*  *Internalized and anticipated stigma among participants*  Study perceptions  *Mistrust, confidentiality concerns*  *Misperceptions of the study aims*  *Apprehensions around study medication*  Study eligibility criteria  *Exclusion related to economic status (lack of phone, housing, transportation)*  *Exclusion related to “vulnerability” (criminal-legal system involvement)*  *Other exclusion criteria (e.g., drug use patterns, comorbidities)* |
| ***Facilitators*** |
| Establishing trust  *Distinguishing research from clinical care*  *Utilizing staff with lived experience*  *Emphasizing transparency, voluntariness*  Referral sources  *Establishing in-house referral systems*  *Creative, community-specific outreach strategies*  *Recruitment and retention specialists* |
| **Retention** |
| ***Barriers*** |
| Socioeconomic marginalization and inflexible protocols  *Transportation to study visits*  *Housing instability*  Scheduling and staffing  *Varying schedules of participants, restricted study hours*  *Limited staff capacity*  *Staff turnover* |
| ***Facilitators*** |
| Building a positive research environment  *Maintaining relationships over time*  *Study stipends*  *Providing amenities in welcoming spaces*  Incorporating flexibility  *Bringing the study to the participant, when necessary*  *Strategies to address transportation barriers* |
